# Demospongic Acids Revisited

**DOI:** 10.3390/md8102569

**Published:** 2010-10-08

**Authors:** Jean-Michel Kornprobst, Gilles Barnathan

**Affiliations:** Groupe Mer, Molécules, Santé MMS/EA 2160, Equipe 7: Lipides marins à activité biologique, Pôle Mer et Littoral, Faculté de Pharmacie, Université de Nantes, France; E-Mail: gilles.barnathan@univ-nantes.fr

**Keywords:** demospongic acids, marine lipids, sponges, marine invertebrates, non-methylene interrupted fatty acids

## Abstract

The well-known fatty acids with a Δ5,9 unsaturation system were designated for a long period as demospongic acids, taking into account that they originally occurred in marine Demospongia sponges. However, such acids have also been observed in various marine sources with a large range of chain-lengths (C_16_–C_32_) and from some terrestrial plants with short acyl chains (C_18_–C_19_). Finally, the Δ5,9 fatty acids appear to be a particular type of non-methylene-interrupted fatty acids (NMA FAs). This article reviews the occurrence of these particular fatty acids in marine and terrestrial organisms and shows the biosynthetic connections between Δ5,9 fatty acids and other NMI FAs.

## 1. Introduction

The well-known notion of demospongic acid appeared for the first time in 1976 in a historical paper from Litchfield and Morales [1], but at that time only as «demospongiae fatty acids». In another paper published in 1980 [2], Litchfield *et al.* used the term «demospongic fatty acids» probably for the first time. Since then and up until now [3–5], this term has widely been used. However, about 35 years after Litchfield’s work on sponge lipids, the notion of demospongic acid seems to no longer have significance, mainly due to their controversial definition and to their wide distribution among marine invertebrates and some terrestrial plants. At the time, it seemed to be of interest to precisely identify the function of demospongic acids, in consideration of their biological activities as topoisomerase inhibitors or against cancer cells as recently reviewed [6], whereas the biological interests of terrestrial short-chain Δ5,9 fatty acids (FAs) had already been demonstrated [7,8].

## 2. What Exactly Is a Demospongic Acid?

The definition of a demospongic acid has never been very clear [1,2]. In their first papers, Litchfield *et al*. only mentioned very long-chain C_24_–C_30_ or C_24_–C_34_ acids with the unusual 5,9 unsaturation pattern, but at that time, only fatty acids with an even number of carbons had been found [1,9]. In the 1980s, a lot of work was published on sponge FAs, and it became apparent that “demospongic acids” also contained all odd FAs from C_23_–C_31_ [10]. Within this field of research, a consensus was quickly established that agreed that demospongic acids were very long-chain fatty acids, mainly C_24_–C_30_, with the atypical 5,9-diunsaturation system, independent of the total number of double bonds. Some years later—and due to many papers being devoted to FAs from sponges—it appeared that:

- the distinction between long-chain fatty acids (LCFAs, C_20_–C_22_ ?) and very long-chain FAs (VLCFAs, ≥C_23_ ?) is not clear and often depends on the authors’ interpretation [10–17].
- the presence of the ever-mentioned 5,9-diunsaturation pattern cannot be considered as characteristic of “demospongic acids” due to the elongation process during their biosynthetic pathways, and diunsaturations such as 5,9-, 7,11-, 9,13-, 11,15-, 17,21-, 19,23- 21,25- and 23,27- can be considered as being similar [18], but other dienoic patterns with short chains such as 6,11-18:2 and 6,11-20:2 have also been considered as “demospongic” acids [19]. Furthermore, several “demospongic acids” display *E* and *Z* configurations for Δ5 and Δ9 double bonds [20].

Currently, the best definition for a demospongic acid would be that of Christie [18], stating “*bis-methylene-interrupted cis-double bonds, ranging in chain-length from C_16_* *to C_34_* *with a* *cis, cis-dienoic system, either with the double bonds in position 5 and 9, or derived from 5,9-16:2 by chain elongation*”.

At present, the question is whether such acids are not at all specific to demosponges, but have been found in other groups of sponges, especially among hexactinellida, in different phyla of marine invertebrates and in several species of terrestrial plants, especially conifers, and in some species of Apocynaceae, Malvaceae, and Ranunculaceae.

## 3. Occurrence of “Demospongic” Acids among Other Organisms

Table 1 presents a non-exhaustive list of more than 40 FAs that correspond to Christie’s definition of demospongic acids found in microorganisms, marine invertebrates and terrestrial plants. A particularly interesting point is the presence of 6-Br-5,9-FAs that are very common in demosponges but quite rare in other organisms. To the best of our knowledge only some Cnidaria Hexacorallia were shown to contain these brominated FAs [21–23], which prove the existence of bromoperoxidases in this group of Cnidaria since it has been proved that these brominated demospongic acids are synthesized by the sponge itself in the final stage of biosynthesis [24].

## 4. Towards a Classification of Non-Methylene-Interrupted Fatty Acids?

**Figure 1:**
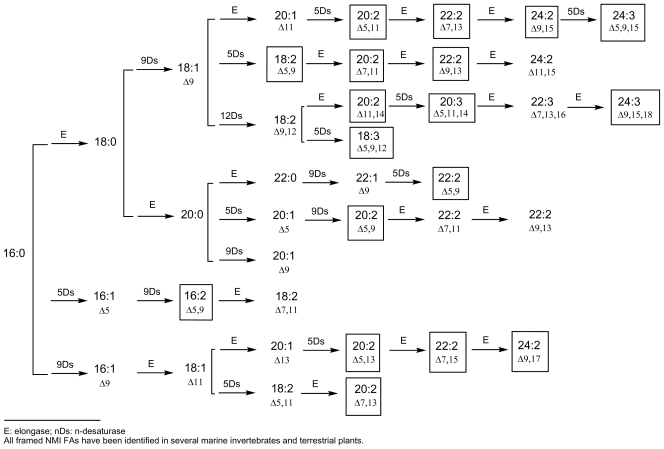
Currently accepted pathways for the main long-chain NMI FAs (≤C_24_) E: elongase; nDs: n-desaturase All framed NMI FAs have been identified in several marine invertebrates and terrestrial plants.

**Figure 2:**
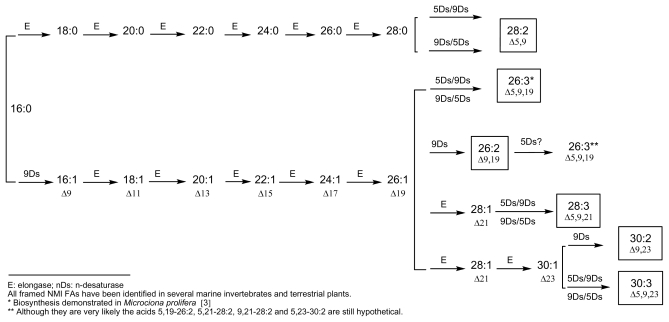
Currently accepted pathways for the main very long-chain NMI FAs (>C_24_) E: elongase; nDs: n-desaturase All framed NMI FAs have been identified in several marine invertebrates and terrestrial plants. *Biosynthesis demonstrated in *Microciona prolifera [*3] **Although they are very likely the acids 5,19-26:2, 5,21-28:2, 9,21-28:2 and 5,23-30:2 are still hypothetical.

Demospongic acids represent a particular type of non-methylene-interrupted FA and, according to Christie’s definition, it could be interesting to consider at least three classes of non-methylene-interrupted fatty acids (NMI FAs) depending on the number of methylene groups situated between the two first double bonds. Then, group 1 would contain all *bis*-methylene-interrupted *cis-*double bonds and would correspond to the series 5,9; 7,11; 9,13… dienoic or polyenoic acids (demospongic acids). Group 2 would be that of *tetra*-methylene-interrupted *cis-*double bonds and would contain the series 5,11; 7,13; 9,15… NMI FAs, such as the acids 7,13-20:2 found in the Brittle star (Echinoderm, Ophiuroidea) *Ophiura sarsi* [37] and in the maritime pine *Pinus pisaster* [29], or the acid 7,13-22:2 found in the sponge *Petrosia ficiformis* [38]. Finally, group 3 would contain *hexa*methylene-interrupted *cis-*double bonds corresponding to the series 5,13; 7,15; 9,17… NMI FAs, such as the acid 7,15-20:2 found in the sponge *Dysidea fragilis* [39]. Some other acids of these three groups have been identified in marine invertebrates, especially molluscs and arthropods, and in numerous terrestrial plants, especially gymnosperms, and all of them can be deduced from accepted biosynthetic pathways implying elongases and 5- and 9-desaturases. Figures 1 and 2 give an overview of these putative biosyntheses from palmitic acid (16:0), palmitoleic acid (9-16:1) and linoleic acid (9,12-18:2). These schemes are currently used and have appeared recently in several publications, along with recent reviews on elongases and polyketide synthases [3,9,10,12,40–44].

## 5. Conclusion

To end this point of view, we think that the former notion of demospongic acid should no longer be used mainly because *bis*-methylene interrupted 5,9-diunsaturated FAs and related acids are distributed among several phyla of marine organisms and several classes of terrestrial plants. The former “demospongic acids” can be considered as a particular series of NMI FAs produced by different combinations of elongases and Δ5 and Δ9 desaturases on the most common FAs in nature such as palmitic and palmitoleic acids.

## Figures and Tables

**Table 1:** Occurrence of “demospongic”* acids in organisms that are not demosponges.

Acids	Genera/species	Kind of organisms	Ref.
5,9-16:2	*Stoichactis helianthus*	Cnidaria (Hexacorallia)	[22]

5,9-17:2	*Dictyostelium discoideum*	Microorganism, soil-living amoeba	[25]

5,9-18:2 taxoleic acd	*Condylactis gigantea*, *Palythoa caribaeorum*, *Stoichactis helianthus*	Cnidaria (Hexacorallia)	[22,23]
	*Cellana grata*, *Collisella dorsuosa*	Marine molluscs	[26,27]
	*Tripneustes esculentus*	Echinoderm	[28]
	*Ginkgo biloba*	Terrestrial plant	[7]

5,9,12-18:3 pinolenic acid (*Z*,*Z*,*Z*) and/or columbinic acid(*E*,*Z*,*Z*)	*Abies* sp., *Cedrus* sp., *Cupressus* sp., *Juniperus* sp., *Laryx* sp., *Picea* sp., *Pinus* sp., *Sequoia* sp., *Thuya* sp.	Terrestrial plants (conifers, gymnosperms)	[8,29]
	*Anemone leveillei* (Ranunculaceae)		[30]

5,9,12,15-18:4	*Perna canaliculus*	Marine mollusc (Lamellibranchiata)	[31]
	*Abies* sp., *Cedrus* sp., *Cupressus* sp., *Juniperus* sp., *Laryx* sp., *Picea* sp., *Sequoia* sp., *Thuya* sp.	Terrestrial plants (conifers)	[8,29]

5,9-19:2	*Allamanda cathartica* (Apocynaceae) *Malvaviscus arboreus* (Malvaceae)	Terrestrial plants (angiosperms)	[32]

*i*-5,9-19:2	*Allamanda cathartica* (Apocynaceae) *Malvaviscus arboreus* (Malvaceae)	Terrestrial plants (angiosperms)	[32]

*ai*-5,9-19:2	*Allamanda cathartica* (Apocynaceae) *Malvaviscus arboreus* (Malvaceae)	Terrestrial plants (angiosperms)	[32]

5,9,12,16-19:4	*Perna canaliculus*	Marine mollusc (Lamellibranchiata)	[31]

5,9-20:2	*Condylactis gigantea*, *Palythoa caribaeorum*, *Stoichactis helianthus*	Cnidaria (Hexacorallia)	[22,23]

6-Br,5,9-20:2	*Condylactis gigantea*, *Palythoa caribaeorum*	Cnidaria (Hexacorallia)	[22,23]

7,11-20:2	*Penaeus setiferus*	Arthropod (shrimp)	[33]

5,9-21:2	*Condylactis gigantea*, *Stoichactis helianthus*	Cnidaria (Hexacorallia)	[22,23]

6-Br,5,9-21:2	*Stoichactis helianthus*	Cnidaria (Hexacorallia)	[22]

5,9,12,15,18-21:5	*Perna canaliculus*	Marine mollusc	[23]

5,9-22:2	*Condylactis gigantea*, *Palythoa caribaeorum*, *Stoichactis helianthus*	Cnidaria (Hexacorallia)	[22,23]
	*Cellana grata*, *Collisella dorsuosa*	Marine molluscs	[26–27]

6-Br,5,9-22:2	*Stoichactis helianthus*	Cnidaria (Hexacorallia)	[22]

9,13-22:2	*Penaeus setiferus*	Arthropod (shrimp)	[33]

5,9,15-22:3	*Collisella dorsuosa*	Marine molluscs	[27]

5,9,19-22:3	*Stoichactis helianthus*	Cnidaria (Hexacorallia)	[22]

5,9-23:2	*Stoichactis helianthus*	Cnidaria (Hexacorallia)	[22]

5,9-24:2	*Condylactis gigantea*, *Palythoa caribaeorum*	Cnidaria (Hexacorallia)	[23]
	*Cellana grata*, *Chromodoris* sp., *Collisella dorsuosa*, *Phyllidia coelesti*	Marine molluscs	[26,27,34]

5,9,15-24:3	*Cellana grata*, *Collisella dorsuosa*	Marine molluscs	[26,27]

5,9,17-24:3	*Cellana grata*, *Collisella dorsuosa*	Marine molluscs	[26,27]

5,9,15,18-24:4	*Cellana grata*	Marine mollusc	[26]

5,9,15,18,21-24:5	*Cellana grata*	Marine mollusc	[26]

5,9-25:2	*Chromodoris* sp., *Phyllidia coelesti*	Marine molluscs	[34]
	*Bebryce studeri*	Cnidaria (Octocorallia)	[35]

*i*-5,9-25:2	*Phyllidia coelesti*	Marine molluscs	[33]

5,9-26:2	*Heterochone* sp.	Marine sponge, Hexactinellida Marine molluscs	[36]
	*Chromodoris* sp., *Phyllidia coelesti*	Cnidaria (Octocorallia)	[34]
	*Bebryce studeri*		[35]

*i*-5,9-26:2	*Chromodoris* sp., *Phyllidia coelesti*	Marine molluscs	[34]

5,9,19-26:3	*Bebryce studeri*	Cnidaria (Octocorallia)	[35]

5,9-28:2	*Aulosaccus* cf. *mitsukuri*, *Heterochone* sp., *Rosella* sp., *Sympagella nux*	Marine sponges, Hexactinellida Cnidaria (Octocorallia)	[36]
	*Bebryce studeri*		[35]

5,9,19-28:3	*Bebryce studeri*	Cnidaria (Octocorallia)	[35]

5,9,23-28:3	*Hyalonema* sp.	Marine sponge, Hexactinellida	[36]

5,9-29:2	*Hyalonema* sp.	Marine sponge, Hexactinellida	[36]

5,9,22-29:3	*Acanthascus* sp., *Aulosaccus* cf. *mitsukuri*, *Euplectella* sp., *Heterochone* sp., *Hyalonema* sp.	Marine sponges, Hexactinellida	[36]

5,9,21-30:3	*Acanthascus* sp., *Aulosaccus* cf. *mitsukuri*, *Euplectella* sp., *Hyalonema* sp., *Heterochone* sp., *Staurocalyptus* sp., *Sympagella nux*	Marine sponges, Hexactinellida	[36]

5,9,23-30:3	*Acanthascus* sp., *Aulosaccus* cf. *mitsukuri*, *Euplectella* sp., *Farrea* sp., *Heterochone* sp., *Hyalonema* sp., *Ipheteon panicea*, *Staurocalyptus* sp., *Sympagella nux*	Marine sponges, Hexactinellida	[36]

5,9,25-30:3	*Hyalonema* sp.	Marine sponges, Hexactinellida	[36]

5,9-31:2	*Hyalonema* sp.	Marine sponge, Hexactinellida	[36]

5,9,21-31:3	*Staurocalyptus* sp.	Marine sponge, Hexactinellida	[36]

5,9,22-31:3	*Acanthascus* sp., *Aulosaccus* cf. *mitsukuri*,	Marine sponges, Hexactinellida	[36]

5,9,23-32:3	*Ipheteon panicea*, *Staurocalyptus* sp.	Marine sponges, Hexactinellida	[36]

* According to Christie’s definition.
